# Spatio-temporal epidemiology of anthrax in *Hippopotamus amphibious* in Queen Elizabeth Protected Area, Uganda

**DOI:** 10.1371/journal.pone.0206922

**Published:** 2018-11-28

**Authors:** Margaret Driciru, Innocent B. Rwego, Benon Asiimwe, Dominic A. Travis, Julio Alvarez, Kimberly VanderWaal, Katharine Pelican

**Affiliations:** 1 Queen Elizabeth National Park, Uganda Wildlife Authority, Kampala, Uganda; 2 Department of Biosecurity, Ecosystems and Veterinary Public Health, College of Veterinary Medicine, Animal Resources and Biosecurity, Makerere University, Kampala, Uganda; 3 Department of Veterinary Population Medicine, College of Veterinary Medicine, University of Minnesota, St. Paul, Minneapolis, United States of America; 4 Department of Medical Microbiology, College of Health Sciences, Makerere University, Kampala, Uganda; 5 VISAVET Health Surveillance Center, Universidad Complutense, Madrid, Spain; 6 Departamento de Sanidad Animal, Facultad de Veterinaria, Universidad Complutense, Madrid, Spain; Spectrum Health, UNITED STATES

## Abstract

**Background:**

Anthrax is a zoonotic disease primarily of herbivores, caused by *Bacillus anthracis*, a bacterium with diverse geographical and global distribution. Globally, livestock outbreaks have declined but in Africa significant outbreaks continue to occur with most countries still categorized as enzootic, hyper endemic or sporadic. Uganda experiences sporadic human and livestock cases. Severe large-scale outbreaks occur periodically in hippos (*Hippopotamus amphibious)* at Queen Elizabeth Protected Area, where in 2004/2005 and 2010 anthrax killed 437 hippos. Ecological drivers of these outbreaks and potential of hippos to maintain anthrax in the ecosystem remain unknown. This study aimed to describe spatio-temporal patterns of anthrax among hippos; examine significant trends associated with case distributions; and generate hypotheses for investigation of ecological drivers of anthrax.

**Methods:**

Spatio-temporal patterns of 317 hippo cases in 2004/5 and 137 in 2010 were analyzed. QGIS was used to examine case distributions; Spearman’s nonparametric tests to determine correlations between cases and at-risk hippo populations; permutation models of the spatial scan statistics to examine spatio-temporal clustering of cases; directional tests to determine directionality in epidemic movements; and standard epidemic curves to determine patterns of epidemic propagation.

**Key findings:**

Results showed hippopotamus cases extensively distributed along water shorelines with strong positive correlations (p<0.01) between cases and at-risk populations. Significant (*p*<0.001) spatio-temporal clustering of cases occurred throughout the epidemics, pointing towards a defined source. Significant directional epidemic spread was detected along water flow gradient (206.6°) in 2004/5 and against flow gradient (20.4°) in 2010. Temporal distributions showed clustered pulsed epidemic waves.

**Conclusion:**

These findings suggest mixed point-source propagated pattern of epidemic spread amongst hippos and points to likelihood of indirect spread of anthrax spores between hippos mediated by their social behaviour, forces of water flow, and persistent presence of infectious carcasses amidst schools. This information sheds light on the epidemiology of anthrax in highly social wildlife, can help drive insight into disease control, wildlife conservation, and tourism management, but highlights the need for analytical and longitudinal studies aimed at clarifying the hypotheses.

## Introduction

Anthrax is an ancient bacterial zoonotic disease, primarily of herbivores, caused by *Bacillus anthracis*, a rod-shaped, gram-positive, endospore forming, capsulated bacteria [1–5]. *B*. *anthracis* is a ubiquitous soil dweller with a diverse geographical and global distribution majorly determined by presence of high risk soils and environmental conditions that favour survival of spores [6–9]. The disease has greatly declined in developed countries due to successful livestock vaccinations [10]. However, most African countries are still categorised as being in the enzootic, hyper endemic or sporadic anthrax ecological zones [11], with severe outbreaks and significant losses in free ranging wildlife, livestock, and humans [3,10,12–16, 17]. Uganda experiences wide-spread, sporadic outbreaks [11,18] in livestock and humans, and periodic large-scale wildlife outbreaks [19,20]. Anthrax has a long history in wildlife in Queen Elizabeth Protected Area (QEPA) [18–20]. The first available diagnosed record was that of Randall in 1956 [18], but there is evidence to suggest that the disease had been present in the park long before this time [19–20]. Severe outbreaks have since been reported in the common hippopotamus (*Hippopotamus amphibious*) with highly significant losses [3,19–20], but no consistent detailed mortality records existed before a massive outbreak in 2004/5. Over 300 hippos died in 2004/2005 [20], and 137 in a subsequent outbreak in 2010. Gourley’s account in 1956 (18) for a similar outbreak in hippopotamus suggests that many such major historical outbreaks in the parks were left to run their course, given the impossibility of control [18], innevitably with high consequences for public health and ecosystem health management. This study utilized the detailed mortality records for 2004/5 and 2010 outbreaks to assess spatio-temporal epidemiology of wildlife anthrax outbreaks in Uganda. The study findings will aid formulation of hypothesis for in-depth studies to identify drivers of anthrax in the ecosystem, and the information could help drive insight into disease control, wildlife conservation and management.

Wild herbivores are highly susceptible to anthrax and mortality rates reach 21–55.5% in hippos, and up to 90% in other species such as impala and kudu [12,16,21]. Despite this significant impact, wildlife outbreaks are still believed to occur cyclically, acting as natural population reduction measures in susceptible species whose densities exceed carrying capacities [22]. Limited control measures therefore exist, but are costly and complex to implement.

It is a commonly held view that anthrax outbreaks occur as point-source episodes with limited geographical spread, and infections develop in specific hot spot areas where soils are heavily contaminated with spores, rather than spread horizontally to new areas [23–26]. Outbreaks are precipitated by environmental conditions that favour spore germination, sporulation and dissemination or conditions that enhance host infectivity or depress host immunity, like extreme drought and flooding, soils rich in calcium, neutral-to-alkaline pH (5–9), relative humidity exceeding 96% and ambient temperatures ranging from 8°C to 45°C [10, 24,27,28]. Despite being an ancient disease, the epidemiology of anthrax remains complex. Movement of *B*. *anthracis* between outbreaks is still poorly understood [25] and unique patterns of spread that are difficult to explain continue to be reported. A classical example is the 1978–1980 epizootic of Zimbabwe that occurred with an unusually high amount of geographical spread [17,26].

*B*. *anthracis*is a non-invasive pathogen and regarded as non-contagious, direct animal-to-animal transmission is therefore thought not to occur except through osgeophagia or carnivore activities [4,27]. Herbivore infections arise indirectly through inhalation or uptake of spores from the environment rather than by contact [3,4,10,27,29]. Carnivory in hippos has lately been reported responsible for high mortalities during anthrax outbreaks [30].

The common hippopotamus is a vulnerable but not endangered wildlife species [31], but forms a significant component of the savannah ecosystem, and contributes > 50% of the total herbivore biomass and standing crop in Queen Elizabeth National Park (QENP) [32–34]. Historically, QENP has held the highest population density of hippos in the world [31,32,34,35]. This is a key factor in ecological processes of habitat modification and ecosystem health as the grazing behaviour impacts plant standing crop, and is a determinant for changes in plant species abundance, composition, and succession [32–36]. Hippos graze by pulling off grass with tussocks, this specifically exposes the soil and induces formation of bare patches in grasslands that become prone to erosion [33,35,36]. *Bacillus anthracis* spores lodge in grass plant roots, as an adaption for spores to increase the likelihood of infecting ungulate hosts [10,37], and this presumably exposes hippos to ingestion of anthrax spores in the soil or plant roots. Historical anthrax outbreak data [20] show that anthrax mortalities in buffalo (*Synceruscaffer*) follow initial epidemic peaks in hippos while sporadic outbreaks occur in waterbuck (*Kobus ellipsiprymnus*), warthog (*Phacochoerus africanus*), elephants (*Loxodonta africana*) and antelopes. Infrequent cases in neighbouring communities [19,20] are presumed to be spill over from the infected animal carcasses. It is therefore hypothesized that the hippos play a significant role in the dynamics and maintenance of anthrax in the study ecosystem.

Despite its historical impact on wildlife, the eco-epidemiology of this disease is still poorly understood, and the exceptional susceptibility of the hippos is difficult to explain. Spatial and temporal analyses have been used in epidemiology to investigate disease origin, onset, aggregation, progression, transmission dynamics, exposure, and guides targeted control [38–43]. This study used data from two outbreaks to examine significant patterns associated with cases.

## Materials and methods

### Study area

This study was conducted in Queen Elizabeth Protected Area (QEPA), a wildlife ecosystem in south-western Uganda (S1 Fig). This area lies at Longitude 29° 45' and 30° l5' East and Latitude 0° I5' North and 0° 30' South and comprises of Queen Elizabeth National Park (QENP) (1,978km^2^) and two adjoining wildlife reserves, Kyambura (157km^2^) and Kigezi (330km^2^). The site is a Man and Biosphere Reserve (MAB), harbours 11 fishing enclaves which hold over 30,000 people and livestock that share same water sources and pasture with wildlife. MAB sites are terrestrial or coastal marine ecosystems recognized by United Nations Educational, Scientific and Cultural Organization (UNESCO) where people and nature live in a balanced relationship [44,45], but QENP is an endemic area for anthrax and a rather porous human-wildlife-livestock disease interface exists [18–20]. Situated on a Rift Valley floor, soils consist mostly of porous volcanic ash, alluvial and pre-Cambrian rocks, and rift valley sediments rich in phosphorus and calcium with alkaline pH [44,45]. These are soil characteristics recognized as risky and conducive for *B*. *anthracis* spore survival [10, 24,27,28]. With the existing optimal climatic conditions characterized by warm-wet and hot-dry regimes, annual precipitations ranging between 600–1,400 mm and temperatures of 15.1°C—36.1°C [45], anthrax outbreaks regularly flare up at epidemic scale or sporadically [3,18,20] presumably once the triger factors are at threshold.

### Study population

Anthrax in the study area primarily affects the common hippopotamus. Hippos are semi-aquatic large herbivores; that live in water during day in clustered social groups called schools, and graze at night in social cohorts [36,33]. Their aquatic behaviour in schools is characterized by tight cohesiveness and congregation of individuals in enormous clumps in shallow, calm, and low current waters with each hippo resting and supporting its head and body on the other (S2 Fig); a strict dominant hierarchal society is expressed by grooming through licking of the body or anal region of lower ranking or prostrated partners; vicious territorial fights among males inflicts fatal injuries [36]. Currently, QEPA has a population of 6,532 hippos (UWA hippopotamus census report for 2016).

### Study design

Datasets used in this study were retrospectively collected from two independent anthrax outbreaks that occurred five years apart, in 2004/5 and 2010 for which consistent detailed mortality records were available. The 2004/5 data was collected from onset to end of outbreak, from 25^th^ July 2004 to May 2005. Similarly, data for 2010 outbreak was collected from onset to end of outbreak, from June 11^th^ to December 2010. Datasets for animal species (2010 figures in parenthesis) were from 318(137) common hippopotamuses (*Hippopotamus amphibius*); 60(17) Cape buffalo (*Syncerus caffer*); 3(2) defassa waterbuck (*Kobus ellipsiprymnus*); 1(1) African elephant (*Loxodonta africana*); and 12(0) Uganda Kob (*Kobus kobthomasi*). For spatio-temporal and epidemic curve analyses, only the hippo data were used. Cases included in data analysis met a criterion for case definition, case classification and attribute data outlined below.

#### Case definition

A case was defined empirically using guidelines for anthrax case definition in animals provided by Centres for Disease Control and Prevention (CDC), Food and Agricultural Organization of the United Nations (FAO) and World Health Organization (WHO) [4,46], (https://wwwn.cdc.gov/nndss/conditions/anthrax/case-definition/2010/) as: 1) Clinical case definition: sudden death, and a carcass oozing un-clotted tarry blood through the mouth, nose and anus and bloating; 2) Post mortem case definition: dark un-clotted blood and splenomegaly in case of accidental opening; 3) Diagnostic case definition: demonstration of characteristic gram positive, rod shaped, capsulated anthrax bacilli on gram and polychrome methylene blue-stained blood and tissue smears; or confirmation of anthrax by culture or standard PCR techniques at accredited reference laboratories and or at the Uganda National Animal Disease Diagnostic and Epidemiology Center (NADDEC); 4) Additionally, a positive lateral flow Immunochromatographic field test result for *Bacillus anthracis* Protective Antigen (PA) test (United States Navy Medical Research Center) for carcasses showing any signs for clinical case definition was considered.

**Classification of cases:** Cases were classified as either “Suspected” or “Confirmed”. Suspected cases were those with a clinical case definition, and Confirmed cases were those with a diagnostic case definition.

**Inclusion criteria:** all confirmed, plus all suspected cases that occurred during a confirmed outbreak within the same locality and contained at minimum, attribute data for date, coordinates, species, and numbers of cases were included in data analysis. Cases that lacked required attribute data were excluded.

#### Data collection

Case data (numerator) were summarized from 2004/5 [20] and 2010 outbreak data collected by Patrol Rangers (staff of Uganda Wildlife Authority) via a Ranger Based Data Collection (RBDC) system implemented on a daily basis and the data collated in MIST (Management Information Systems) and SMART (Spatial Monitoring and Reporting Tool: http://smartconservationtools.org) designed for monitoring threats to wildlife. Case attribute data included date cases detected, geo-referenced Geographical Positioning System (GPS) coordinate locations, animal species affected, numbers of animals dead, approximate carcass age and disposal actions taken. Reference population data for at-risk hippos (denominator) was collected by UWA in 2005/2006 and 2010, using the outboard speed boat census techniques of Petrides and Swank [35] or foot patrol counts along river banks.

Data attributes used contained geo-referenced coordinate locations of hippo schools and total numbers per school (S3 and S4 Figs and S2 Table).

### Data analysis

#### Case distribution

Geo-referenced case locations and clusters were mapped using QGIS (Free Software Foundation, Inc., 51 Franklin Street, Boston, USA) to assess their spatio-temporal distribution.

To determine if there was a correlation between the number of cases and the hippo population, geo-referenced locations of hippo schools from populations present during the outbreak years of 2004/2005 and 2010 were mapped in QGIS as “at risk populations”. A buffer of 6 kilometres radius representing the grazing range for hippos [36] was then generated around each hippo school using QGIS geo-processing tools. The buffer was overlaid with 6 x 6 km grid cells generated using vector grids research tools and the grids clipped to the buffered area. Finally, the numbers of cases and of at-risk hippos per grid cell were recorded and correlations evaluated using Spearman's nonparametric test which has no requirement for normality of data, this was implemented in SPSS 16 (IBM, Boulder, CO).

#### Spatio-temporal cluster analysis

The existence of local spatio-temporal clustering of cases in space and time were then evaluated using the retrospective space-time permutation model of the spatial scan statistics [41] on SaTScan 9.1.1 software [47,48]. In this analysis, the study area is scanned for the identification of clusters of cases occurring close in both space and time, and the model determines the significance of the most likely clusters by comparing the number of observed cases occurring within each of the possible scanning windows with the expected number of cases generated in 999 Monte Carlo randomizations of date to the observed locations and dates at a maximum window size of 50% of the population at risk.

The onset-to-end time cluster sequence generated by the model was used to reflect the incubation period of the infection [49]. This was compared against referenced incubation periods of anthrax in naturally infected herbivores of 1–14 days on average, 20 days maximum recommended by OIE for trade and control of anthrax [3,4]. Clusters that spanned over 20 days were considered protracted single outbreaks beyond anthrax incubation period.

#### Spread and propagation

The existence of a significant trend on the direction of movement of the epidemic was assessed with the directional test statistics on the ClusterSeer software (TerraSeer, Crystal Lake, IL, USA) using a relative time connection matrix and week (7 days) as the time-step unit [48]. In the relative-directional matrix, the model builds a vector line that connects each case with all other subsequent cases. A measure of the average direction of the vector lines and its angular variance (angular concentration) is then used to estimate the average direction of detection of the movement of the epidemic. If the outbreak follows a consistent trend towards a given direction, then the angular variance is expected to be small, and the angular concentration (restricted to the range 0–1) is large. The level of significance was determined by comparing the observed concentration to an expected value, generated by switching the location and dates of occurrence of the cases through 999 Monte-Carlo simulations.

To examine patterns of epidemic propagation, standard epidemic curves were built using plots of weekly number of cases over time [42], analysed in Microsoft Excel Windows 2010 version 1709.

### Ethical statements for the research project

This study forms part of a bigger research project on ecology of anthrax at the study site. Research approvals were obtained from Uganda National Council for Science and Technology (UNCST) (Ref: NS 418); and Uganda Wildlife Authority (UWA) (Ref: UWA/TDO/33/02) for research in Protected Areas involving wildlife. Ethical approvals, research protocols, tools and bio-safety considerations that have been used for other components of the research project were reviewed and approved by two Institutional Review Boards (IRB) and Research Ethics Committees from College of Health Sciences, School of Medicine (#REC REF 2013–084); and College of Veterinary Medicine, Animal Resources and Biosecurity (COVAB), School of Veterinary Medicine (VAB/BRC/14/101) of Makerere University, Kampala, Uganda.

Data used for 2004/5 outbreak was collected by UWA patrol rangers based on a management oriented protocol called Ranger Based Data Collection System (RBDC). The RBDC is a system designed to collect data for monitoring threats to wildlife and Protected Areas in Uganda, its outputs inform threat reduction management decisions. Data for 2010 was collected by the principal author and patrol rangers using the same protocol.

### Potential limitations of the study

Potential limitations that could influence the data quality may include data collection procedures and reporting practices like the degree of case detection, case definitions and analytical uncertainties. Detection success could have been affected by difficult terrain; vegetation cover; vastness of the national park; patrol planning and execution styles; ability of patrol teams to spot cases; poachers’ or scavenger activities that remove carcasses; and data recording biases or errors. However, reconnaissance surveillance methods used during both outbreaks included daily marine and ranger foot patrols occasionally supported by air surveillance, and carcass disposal. Given this frequency of surveillance and carcass disposal, the authors believe the case detection success was well over 70%. A similar detection probability has been reported for detection of marine bird carcasses assessed using a combination of methods (www.marineornithology.org/PDF/37_3/37_3_197-204.pdf). Information on detection probabilities for large wildlife carcasses was not available. Furthermore, different combinations of analytical procedures were employed to examine results; the authors therefore believe that these biases did not significantly influence the study results and conclusions.

## Results

### Spatial distribution

**Fig 1:**
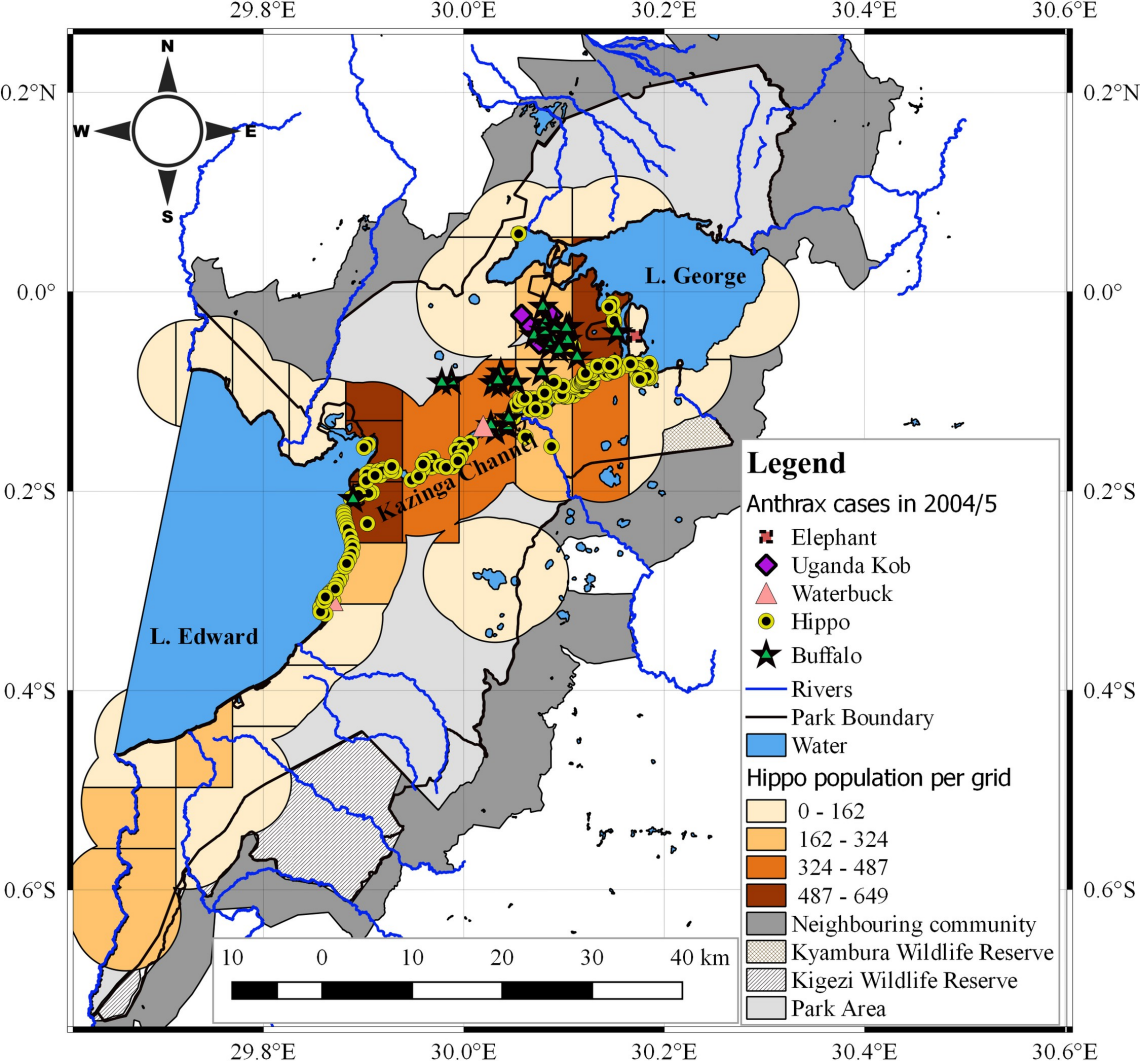
Spatial distribution of anthrax cases relative to hippo population size in 2004/5 outbreak. Symbols represent case locations. Colour ramp from light to dark on gridded rectangle cells represent increasing number of at-risk hippos within a 6 km radius buffer zone. *Hippopotamus amphibious* live in water during day, congregating in social groups called schools. They walk out at night an average of 3–6 km to graze [4]. A buffer of 6 km radius was generated around each geo-referenced school to map high risk areas for hippo anthrax outbreaks. The study area was overlaid with 6 x 6 km grid cells and clipped to the buffers. Twenty-seven (n = 27) grid cells were used to map spatial overlap between cases and hippo populations. Cases were mostly recorded in areas with at-risk hippo populations exceeding 324.

**Fig 2:**
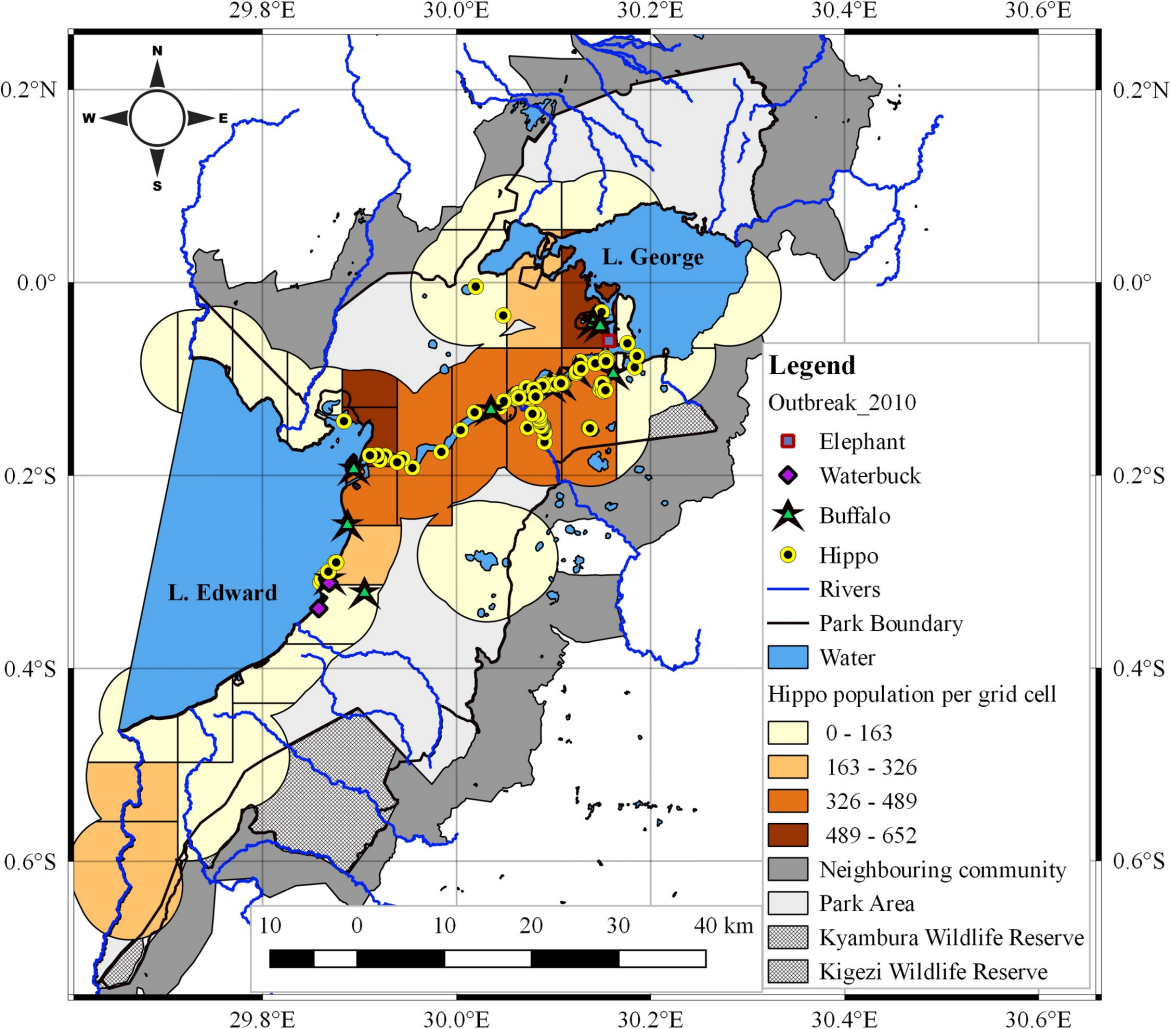
patial distribution of anthrax cases relative to hippo population size in 2010.

*Hippopotamus* anthrax cases in 2004/5 extensively occurred within the central parts of the study area, primarily along shorelines of major water bodies of southern Lake George, eastern Lake Edward, Kazinga channel, River Kyabmura or on inland mud and water pools inhabited by hippos (Figs 1 and S1). The distribution in 2010 was most predominant in the northern half of Kazinga channel (Fig 2). Cases of buffalo (60/17), elephant (1/1), waterbuck (3/2) and Uganda kob (12/0) were recorded within hippo grazing zones in 2004/5 and 2010 outbreaks respectively. Spatial overlap was detected between the number of cases and large population sizes of hippos, especially exceeding 324 per grid cell (Figs 1 and 2). No cases occurred south of the study area.

Strong positive correlations (p<0.01) were detected between the number of recorded anthrax cases and at-risk hippopotami populations per grid cell in 2004/5 (r_2_ = 0.692) and 2010 (r_2_ 0.638) outbreaks (Table 1).

**Table 1:** Spearman’s bivariate correlations for linear associations between hippo cases and population size in 2004/5 and 2010 outbreaks.

Variable	Outbreak Year	Population size		Number of cases	
		2004/5	2010	2004/5	2010
Population size	2004/5	1.000	0.958**	0.692**	0.575**
	2010	0.958**	1.000	0.717**	0.638**
Number of cases	2004/5	0.692**	0.717**	1.000	0.693**
	2010	0.575**	0.638**	0.693**	1.000

**. Correlation was significant at the 0.01 level (2-tailed). Variables were measured per grid cell of size 6km x 6km for n = 27

### Spatio-temporal clusters

**Fig 3:**
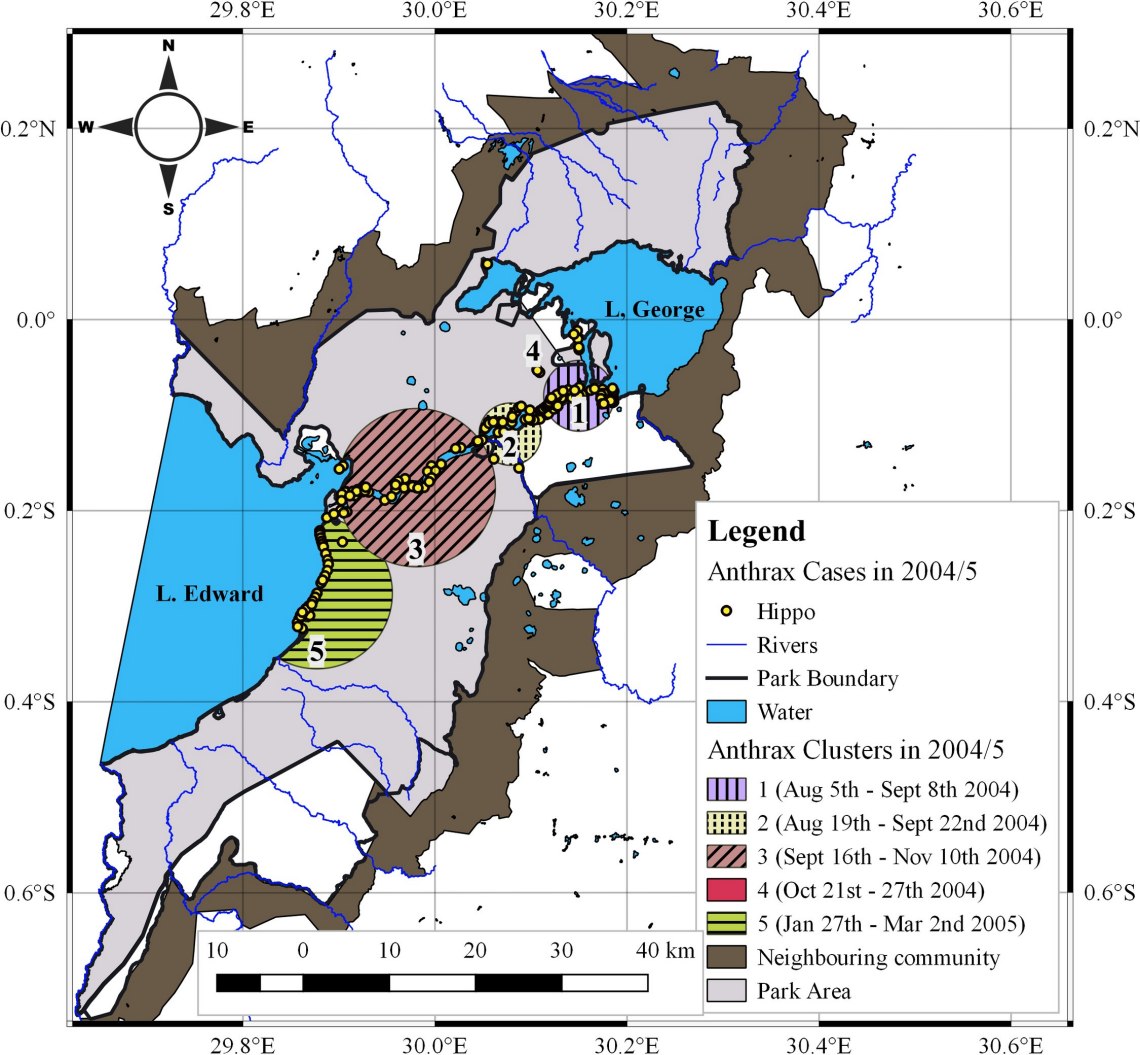
Spatial and temporal distribution of *Hippopotamus* anthrax clusters in 2004/5. Spatio-temporal data for clusters including centroid coordinates, radii, and time span were generated using the retrospective space-time permutation model of the spatial scan statistics and mapped using QGIS (Version 2.18.9). The first two clusters 1 & 2 had overlapping dates and were considered origins for this outbreak.

**Fig 4:**
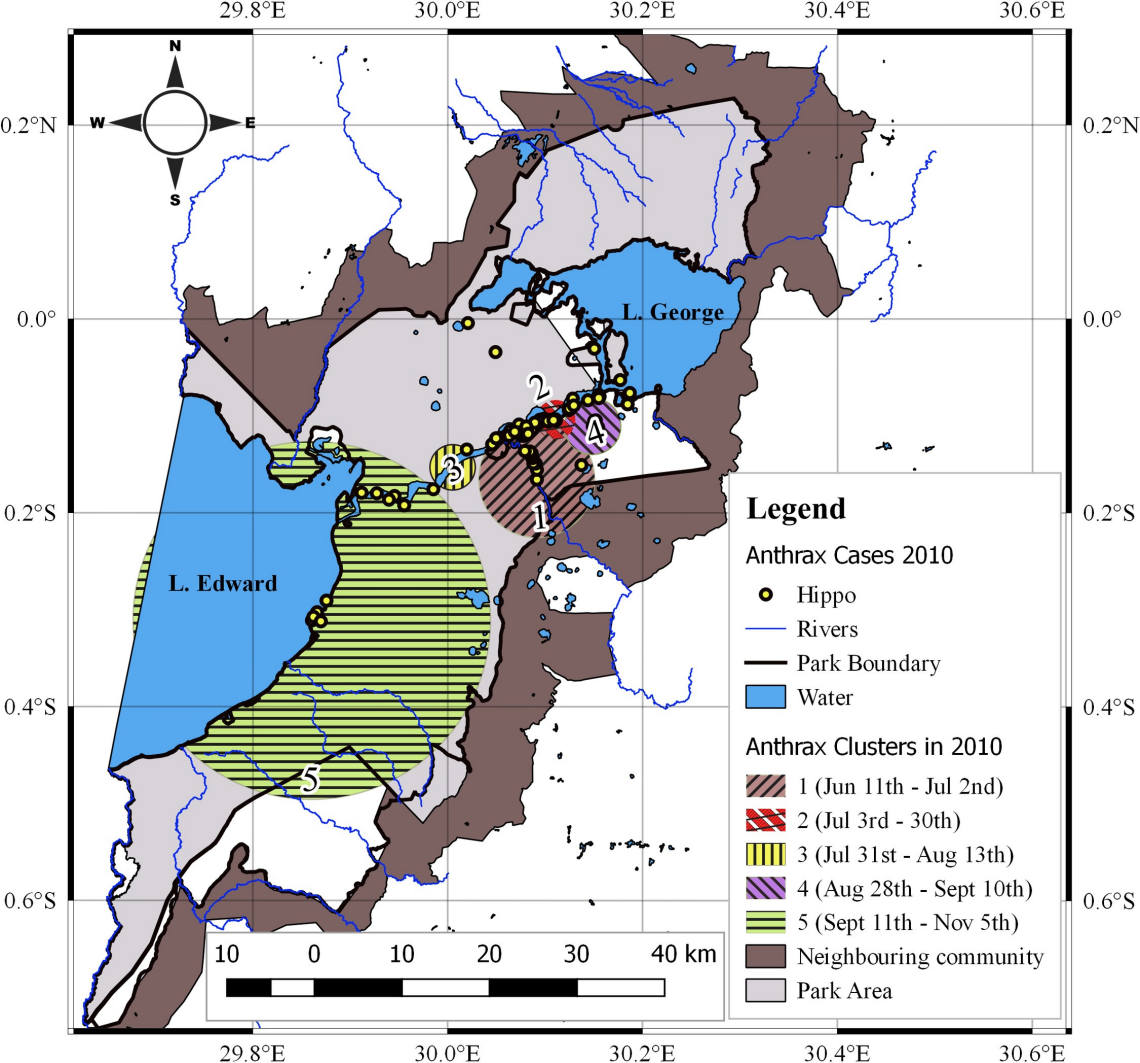
Spatial and temporal distribution of *Hippopotamus* anthrax clusters in 2010. Clusters 2 & 4 occurred upstream, relative to cluster 1 and against direction of water flow.

Significant spatio-temporal clustering of cases was detected throughout both epidemics (*p*<0.001). In 2004/5 (Table 2 and Fig 3), five significant clusters were detected, comprising 18–69 cases. Cluster radii varied from 0.43–9.17 km and spanned 7–55 days. Overlapping dates, reflecting simultaneously occurrence of cases at two separate locations were detected for two clusters (1 & 2) at the onset of this outbreak. In the 2010 outbreak (Table 3 and Fig 4), five significant clusters were detected as well, comprising 4–58 cases. Cluster radii ranged from 2.25–20.43 km and spanned 14–56 days. Protracted outbreaks lasting longer than referenced incubation periods of anthrax were detected for four clusters (1, 2, 3 & 5) in 2004/5 and three clusters (1, 2 & 5) in 2010.

**Table 2:** Temporal characteristics of case clusters in *Hippopotamus* anthrax outbreaks of 2004/5.

Cluster ID	Outbreak time sequence (2004/5)	Outbreak Days	Number of Cases			Radius (km)	*P*—Value
			Observed (O)	Expected (E)	Ratio (O:E)		
1	Aug 5th—Sep 8^th^	35	69	26.21	2.63	4.10	<0.0001
2	Aug 19th—Sep 22^nd^	34	37	11.30	3.27	3.61	<0.0001
3	Sep 16th—Nov 10^th^	55	43	15.80	16.98	9.17	<0.0001
4	Oct 21^st^ - 27^th^	7	18	1.07	16.76	0.43	<0.0001
5	Jan 27th—Mar 2^nd^	34	52	11.20	4.64	8.76	<0.0001

**Table 3:** Temporal characteristics of case clusters in *Hippopotamus* anthrax outbreaks of 2010.

Cluster ID	Outbreak time sequence (2010)	Days of outbreak	Number of Cases			Radius (km)	*P*—Value
			Observed (O)	Expected (E)	Ratio (O:E)		
1	Jun 11^th^—July 2^nd^	21	58	28.90	2.01	6.61	<0.0001
2	July 3^rd^–July 30^th^	27	18	3.95	4.56	2.25	<0.0001
3	July 31st—Aug 13^th^	14	4	0.15	26.66	2.59	0.0011
4	Aug 28^th^—Sep 10^th^	14	10	0.99	10.08	3.21	<0.0001
5	Sept 11^th^—Nov 5^th^	56	16	3.32	4.81	20.43	<0.0001

The spatial and temporal distribution of clusters identified in 2004/5 followed the direction of water flow, beginning with the earliest cluster in L. George (cluster 1), followed in sequence by Kazinga Channel (clusters 2 & 3) and finally L. Edward (cluster 5) (Figs 3 and S1). Only one cluster (4) occurred inland at water pools. The first two clusters (1 & 2) had overlapping dates.

In 2010 (Fig 4), clusters aggregated mostly in the upper half of Kazinga channel. The first (cluster 1) occurred midway in Kazinga channel spanning across its confluence with R. Kyambura. The second and fourth clusters were detected upstream towards L. George, moving against the direction of water flow, but the subsequent clusters 3 & 5 occurred downstream ending in L. Edward. A spatial overlap of clusters between 2004/5 and 2010 outbreaks was observed.

### Spread and propagation

Significant directional movements were detected during both outbreaks on the directional test. In 2004/5, the average direction of spread of the epidemic was 206.6° (pointing Southwest) with an angular concentration of 0.55 (*P* = 0.001). In 2010 the average direction of spread was 20.4° (Northeast) with an angular concentration of 0.11 (*P* = 0.001). These directions connect the clusters occurring early and late in both 2004/5 and 2010 epidemics.

**Fig 5:**
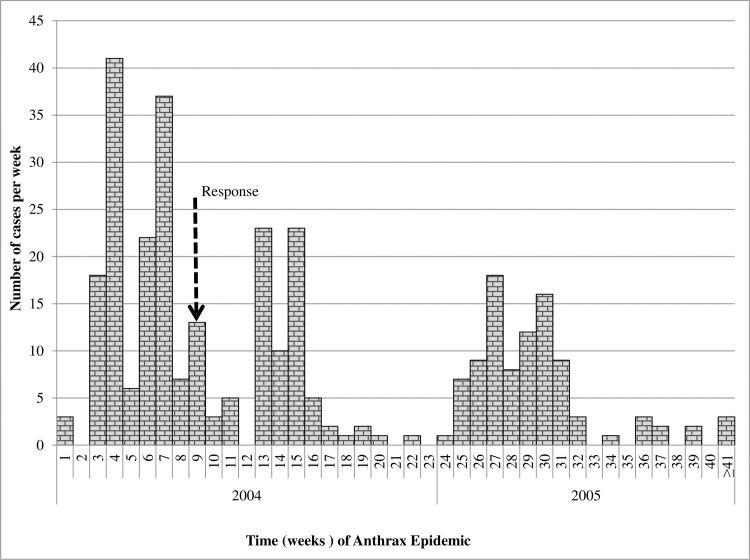
Epidemic curve showing patterns of epidemic spread for weekly anthrax cases in *Hippopotamus*. This outbreak occurred in clustered waves (epidemic generations), lasted 42 weeks from 25 July 2004 to May 2005 and died after 3 generations. Clusters that spanned over 20 days were considered protracted single outbreaks beyond anthrax incubation period or multiple outbreaks (Tables 2 and 3). Dashed line shows time in week 9 of outbreak when management intervention for carcass disposal was initiated. Number of cases did not drastically decline but epidemic peaks in the second and third epidemic generations dropped below the peak in the first wave instead of becoming successively larger.

**Fig 6:**
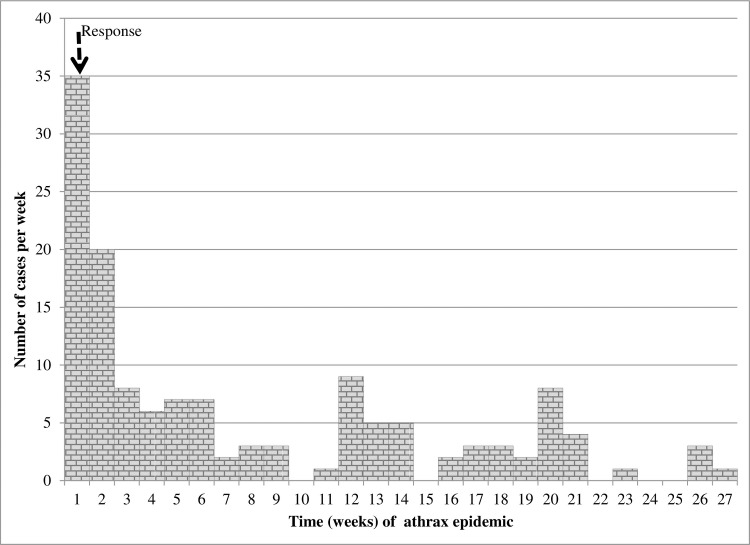
Epidemic curve showing patterns of epidemic spread for weekly anthrax cases in *Hippopotamus* in2010. **O**utbreak occurred in less distinct waves, lasted 27 weeks from 11 June to December and died after 3 generations. Dashed line shows time at onset of the outbreak in week 1 when management intervention for carcass disposal was initiated. Number of new cases drastically declined immediately following response.

**Fig 7:**
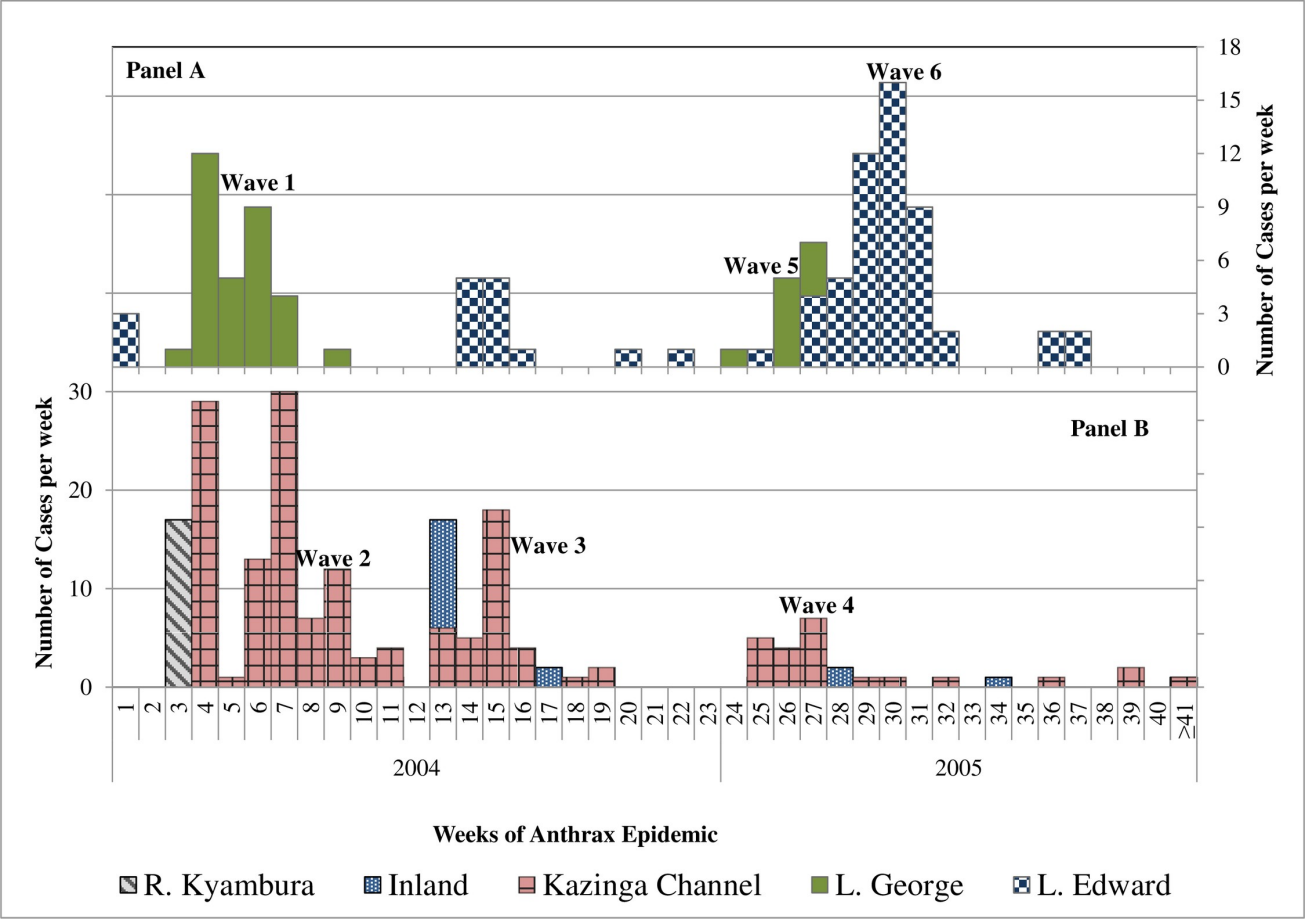
Location specific patterns of epidemic spread for weekly *Hippopotamus* anthrax cases in 2004/5. Epidemic curves were characterized by geographical locations at Lakes George and Edward (Panel A); Kazinga channel and R. Kyambura (Panel B) to examine possibilities of occurrence of a series of sporadic point source outbreaks. Epidemic waves detected were numbered 1–6 according to the sequence of occurrence of waves at the respective locations. Epidemic patterns remained clustered, progressing successively and waves lasted beyond one incubation period of anthrax (average 1–14 days) except at Kyambura River.

Clustered, pulsed multiple epidemic waves (generations) were detected in the epidemic curves during both 2004/5 and 2010 outbreaks (Figs 5 and 6); both epidemics waned after three generations and independent waves showed spiked peaks. The number of cases rose steeply within the first 2 weeks in 2004/5, and within week 1 in 2010; cases in 2010 declined drastically following implementation of carcass disposal response in week 1. In 2004/5, cases did not drop drastically following response action in week 9, but the second and third epidemic peaks dropped below the peak in the first wave instead of becoming successively larger (Fig 5). Independent epidemic waves lasted 8–9 weeks (56–72 days) in 2004/5 (Fig 5) and 4–9 weeks (28–72 days) in 2010 (Fig 6): this implies in general, cases occurred over more than one incubation period of anthrax (1-14/20 days) (Figs 5 and 6). A total of 17 cases at R. Kyambura occurred in seven days, within one incubation period of anthrax. When epidemics were characterized by geographical location in 2004/5 (Fig 7), six waves were detected; epidemic patterns remained clustered, progressing successively along water flow gradient from Kazinga Channel down to L. Edward. Two outbreaks (waves 1 & 2) at L. George and Kazinga channel respectively occurred at the same timing, as well as three outbreaks (waves 4, 5 & 6) at Kazinga channel, L. George and L. Edward

## Discussion

### Spatio-temporal clustering and case distribution

The strong consistent spatio-temporal patterns observed here in two independent anthrax outbreaks (2004/5 and 2010) five years apart and in the same geographical area is suggestive of a localized source of exposure. This is reflected in the intense clustering detected throughout both epidemics (Figs 3 and 4) whereby initial clusters 1 & 2 in 2004/5; and cluster 1 in 2010 both pointed towards defined foci at the confluence of R. Kyambura, Kazinga Channel and southern parts of L. George. The death of 17 out of a population of 45 at-risk hippos at the foci in 2004/5 and 21 out of 95 in 2010 in 7 days (within one incubation period of anthrax), at the onset of outbreaks suggests the likely location of primary cases for these outbreaks, and a high exposure risk area. The shapes of epidemic curves were characterized by steep upward slopes at the onset (Figs 5 and 6), which reflects a sharp rise in initial number of new cases over a short time span, and a gradual or steep decline in the number of cases. These are epidemic patterns associated with point source outbreaks with a common source of exposure [39,42]. Point-source episodes are features reported in naturally occurring anthrax outbreaks, infections develop in high risk soils heavily contaminated with spores [24–26].

Given the historical existence of anthrax and major outbreaks in QEPA [18–20], and presence of high risk soils of volcanic ash, alkaline pH, high calcium and phosphorus content [44,45], the area is endemic and outbreaks are easily precipitated by extreme weather conditions. Hippos being strict grazers that pull off grass with tussocks as they feed [33,35,36,44], have a high risk of ingestion of spores in soil and pasture; and on grass plant roots where *B*. *anthracis*is reported to be lodged [37], as an adaptation for the pathogen to increase the likelihood of infecting ungulate hosts [10].

The spatial overlap between clusters of 2004/5 and 2010 outbreaks; and the nature of case (Figs 1 and 2) and cluster (Figs 3 and 4; and S1 Table) distribution is probably influenced by the distribution of population clusters of at-risk hippo schools. Strong positive correlations detected between number of cases and at-risk hippo populations per grid cells (p<0.01; r_2_ = 0.692 for 2004/5 and 0.638 for 2010) (Table 1) may suggest effect of population density at a localized level. The high degree of congregation and tight cohesiveness exhibited amongst individuals in hippo schools [36] (S2 Fig) is believed to decrease the distance between animals, and induce niche-based population density rise according to Kendal’s Population Threshold Theorem [49] which can facilitate transmission of spores during outbreaks. From early 1950 to late 1970s, QENP held the highest population density of hippos in the world [31,32,34,35], and the frequent anthrax outbreaks recorded in hippos during these years [20] may be associated with high densities.

### Spread and propagation

In 2004/5 outbreak, the epidemic spread in a Southwest (206.6°) direction, following the natural water-flow gradient, suggesting a possible water-borne mechanical propagation of infectious carcasses or dispersal of spores. However, in 2010 the direction of spread pointed Northeast (20.4°), against the logical flow gradient of water. This can suggest multiple alternative transmission mechanisms facilitating spread or effectiveness of a prompt carcass disposal response implemented in week one of 2010 outbreak (Fig 6) curtailed the transmission dynamics.

The epidemic patterns depicted during both outbreaks comprising of clustered, multiple epidemic waves (generations) that waned after three generations, spiked epidemic peaks, occurrence of cases over more than one incubation period of anthrax (Figs 5–7 and Tables 2 and 3) are characteristics described of propagated epidemics [27,39,42,49,50]. Sometimes, a series of point-source epidemics occur that can be difficult to differentiate from propagating epidemics [49]. To further examine, we characterized the epidemic waves by geographical location, but the patterns remained quite distinct (waves 1–6, Fig 7) except for cases occurring simultaneously at different locations.

Transmission in propagated epidemics primarily occurs by direct host-to-host contact [39,42,49]. However, *B*. *anthracis*is is a non-invasive and non-contagious pathogen that exhibits little or no potential for direct animal-to-animal transmission [4, 26, 27]. Although animal-to-animal transmission through osgeophagia, consumption of infected meat, carnivore activities or vectorborne transmission for instance through the bites of tabanidae anthrax vectors (horse flies) have caused notable anthrax outbreaks [4,51,52], these may not substantially account for such consistent wide extent of spread observed here. The commonly held view is that herbivore infections occur indirectly through ingestion of spores from contaminated pasture, soils and water [3,4,10,12,24,26,27]. Carcass sites stained with haemorrhagic body fluids and exudates oozing from animals that terminally die of anthrax are heavily laden with *B*. *anthracis* spores [4,13,14]. For instance, a consistent increase in spore production from vegetative bacterial cells (sporulation) shed in spilt blood and tissue at carcass sites has been reported to occur up to 4–8 days after host death [14]. Given the saltiness of blood, crave of herbivores for salty licks and the associated growth of lush pasture therein, these sites offer primary foci of infection for grazing and craving herbivores [3,4]. The risk of spore ingestion for hippos is presumably high given that they are strict grazers that pull off grass with tussocks while grazing [36]. But the extend and rapid spread observed in this study amongst the hippos and the occurrence of cases primarily on water bodies (Figs 1 and 2) may suggest additional alternative mechanisms of spread.

Gourlay in 1956 [18] similarly reported all hippo carcases occurring on water and large hippo pools where population densities were high. Gourlay, suggested that the mortality patterns were indications that hippos suffered sub-clinical form of anthrax rather than the acute or peracute form reported in ruminants [3], since there were sick hippos observed with oedema on the neck, and the sick presumably had enough time to return from grazing on land to die in water. But given that hippos spend their entire day in those high density schools in water and only 3–6 hours grazing on land [36], coupled with the fact that septicaemic animals seek water, there is a likelihood that infected hippos could get confined to their schools till death. The potential of these microenvironments as spore concentration points cannot therefore be ruled out, especially during outbreaks when infectious carcasses are littered amidst schools for extended periods of time [20].

Airborne transmission (inhalation) readily facilitates rapid spread of specific pathogens [38], and anthrax spores are well adapted to aerosolization [26], especially in humans [53] resulting in severe fatal infections. Although herbivore inhalation infections are believed to be rare, pulmonary anthrax in cattle [4] and infections due to inhalation of spores by animals grazing over dry, dusty, contaminated soils [27] have been reported. To our knowledge, consistent clinical observations of respiratory distress, manifested as ‘gasping for breath’, indicative of pulmonary anthrax have been reported in some terminally ill anthrax infected hippos in four outbreaks at the study area in 1960, 1969, 2010, and 2004/5 [18,20]. This might suggest spore inhalation exposure risks for hippos either on the rangeland or on water within the high-density schools, presumably due to breathing in of bloody water aerosols or sniffing of terminally ill or dead cohorts [4].

Given that anthrax is not transmitted by host-to-host contact, we hypothesize non-mutually exclusive alternative theories to support the propagated epidemic patterns observed in this study:

- *The Intrinsic and classical social behaviour of hippos increases contact between host and B*. *anthracis spores from infectious carcasses littered amidst schools for extended periods of time*. The intense spatio-temporal clustering during both outbreaks in 2004/5 and 2010 (Figs 3 and 4); spatial overlap of cases with areas of high hippo populations (Figs 1 and 2); and the strong positive correlations between cases and at-risk hippo populations are suggestive of a transmission mechanism facilitated by population density. The clustered, multiple epidemic waves depicted on epidemic curves (Figs 5–7) may indicate spread between or within schools. Given the findings, the high degree of congregation and tight cohesiveness amongst hippos in schools [36] (S2 Fig) may become a potential factor that can reduce distance between animals and induce niche-based population density rise required to facilitate spread within schools according to Kendal’s Population Density Theorem [49]. Coupled with the long presence of infectious carcasses littered amidst schools [20,30], and hippo grooming behavior of licking lower ranking or prostrated partners [36], this might enhance inhalation or ingestion of spores, especially if sick prostrated or dead social cohorts get sniffed or groomed [4]. Carnivory amongst hippos has been observed and reported to be responsible for high mortalities from anthrax [30].
- *Water-borne mechanical propagation of infectious carcasses along a directional flow gradient exposes susceptible hippo schools to B*. *anthracis spores along the path*. The consistent significant clustering and directional movement of the epidemic along water flow gradient in 2004/5, suggests spread of infectious carcasses or spore dispersal facilitated by forces of water [3,4, 8,17,25]. Although in contrast, in 2010 the epidemic followed a northeast direction (20.4°), against the logical direction of water flow, this may be due to additional alternative mechanisms of spread or the effectiveness of a prompt carcass disposal response implemented in week one of 2010 outbreak (Fig 6) curtailed the transmission dynamics. Some but not all of the clusters were still downstream of earlier clusters, suggesting water-borne mechanisms may still be at play.

To our knowledge, hippos typically die amidst social cohorts (schools) in water. In large water bodies the carcass immediately sinks to the bottom of water, bloats, comes afloat within 8–16 hours, and either remains within environs of the school for 4–7 days when it rots, disintegrates and sinks again, or is driven by water currents to various destinations.

Like for the case of contaminated soil and pasture, [3,4,13,14,24,25,27], one would expect the large numbers of infectious carcasses amidst schools [12,20,30] exuding haemorrhagic fluids to contaminate the water with spores [14] but this could not be demonstrated by investigators during 2004/5 outbreak [20]. Available literature suggests that fragile *B*. *anthracis* vegetative forms die spontaneously in water or undergo immediate sporulation to survive and yet the spores rapidly loose viability in water due to the rate at which the blood and haemorrhagic fluids are diluted out [4,6,12,13,23]. More so, if carcasses remain intact the vegetative bacilli die within 3–8 days because of anaerobic putrefactive processes [8,10]. But we believe the carcass events coupled with the behaviour of hippos described above offer enough exposure time between susceptible hosts and infective spores. The dramatic reduction in the number of cases following timely carcass disposal measures implemented in 2010 suggests the potential of these carcasses as a source of infection and the effectiveness in their disposal as a control measure. An alternative theory of hippos inhaling spores directly from sick social cohorts amidst the tight congregation in schools or through breathing in of bloody water aerosols cannot therefore be ruled out.

## Conclusion

Consistent highly significant spatio-temporal clustering of cases and directional epidemic movements were detected occurring in clustered pulsed epidemic waves in two *Hippopotamus* anthrax outbreaks in 2004/5 and 2010. The findings suggest a mixed point-source propagated pattern of epidemic spread amongst the *Hippopotamus*.

We think the intrinsic classical social behaviour of hippos congregating in enormous clumps decreases the distance between animals and induces niche-based population density rise and has potential to facilitate spore transmission. The presence of infectious carcasses littered amidst schools during outbreaks, coupled with the grooming behavior of sniffing and licking social cohorts greatly increases contact with spores. There is likelihood of indirect spread of anthrax spores between or within hippo schools in water from primary to secondary cases mediated by forces of water flow, intrinsic social behavior of hippos, and long presence of infectious carcasses amidst schools.

This information sheds light on the epidemiology of anthrax in highly social wildlife, can help drive insight into disease control, wildlife conservation, and tourism management, but highlights the need for analytical and longitudinal studies aimed at clarifying the hypotheses.

## Supporting information

S1 Fig Map showing study area. comprises of: 1) Queen Elizabeth National Park (1978 km^2^): brown coloured polygon; two adjacent wildlife reserves: 2) Kyambura (157 km^2^): diagonal hatched lines; and 3) Kigezi (330 km^2^): vertical hatched lines. There are two major fresh water lakes: L. George (northward) and Edward (southward) connected by 40km long natural Kazinga channel; the area is drained by a network of rivers. QEPA is a Man and Biosphere Reserve (MAB): there are 11 Fishing enclaves, with an estimated population of ≥300,000 people and approximately 30,000 livestock.
(TIF)

S2 Fig A School of *Hippopotamus amphibious*. picture shows tight cohesiveness and congregation of hippos in enormous clumps with each individual resting and supporting its head and body on the other in shallow, calm, and low current waters
(TIF)

S3 Fig Distribution of at-risk hippopotamus schools per buffered grid cells in 2005/2006. population census conducted from November 2005 to January 2006 in all water bodies within study area. Numbers against hippo schools (filled circles) indicate size of school at each location. School sizes varied from 1–63. Buffers represent average grazing distance for hippos of 6km from their respective schools.
(PNG)

S4 Fig Distribution of at-risk hippopotamus schools per buffered grid cells. population census conducted from January to February 2010 in all water bodies within the study area. Numbers against hippo schools (filled circles) indicate size of school at each location. School sizes varied from 1–70. Buffers represent the average grazing distance for hippos of 6km from their respective schools.
(PNG)

S1 Table Data tables. this table contains data sets used for spatial, temporal and epidemic curve analysis in the study
(XLSX)

S2 Table Data table. this table contains data sets for at-risk hippopotamus populations per grid cells used for correlation analysis. Population data for November 2005 to January 2006 census used for 2004/5 outbreak (July to May); census data for January to February 2010 used for outbreaks of June–December 2010.
(XLSX)
