# Supplementary figures and images for: Spatio-temporal epidemiology of anthrax in Hippopotamus amphibious in Queen Elizabeth Protected Area, Uganda
Source: PLoS One. 2018 Nov 28;13(11):e0206922. doi: 10.1371/journal.pone.0206922 (PMC6261556; doi:10.1371/journal.pone.0206922)

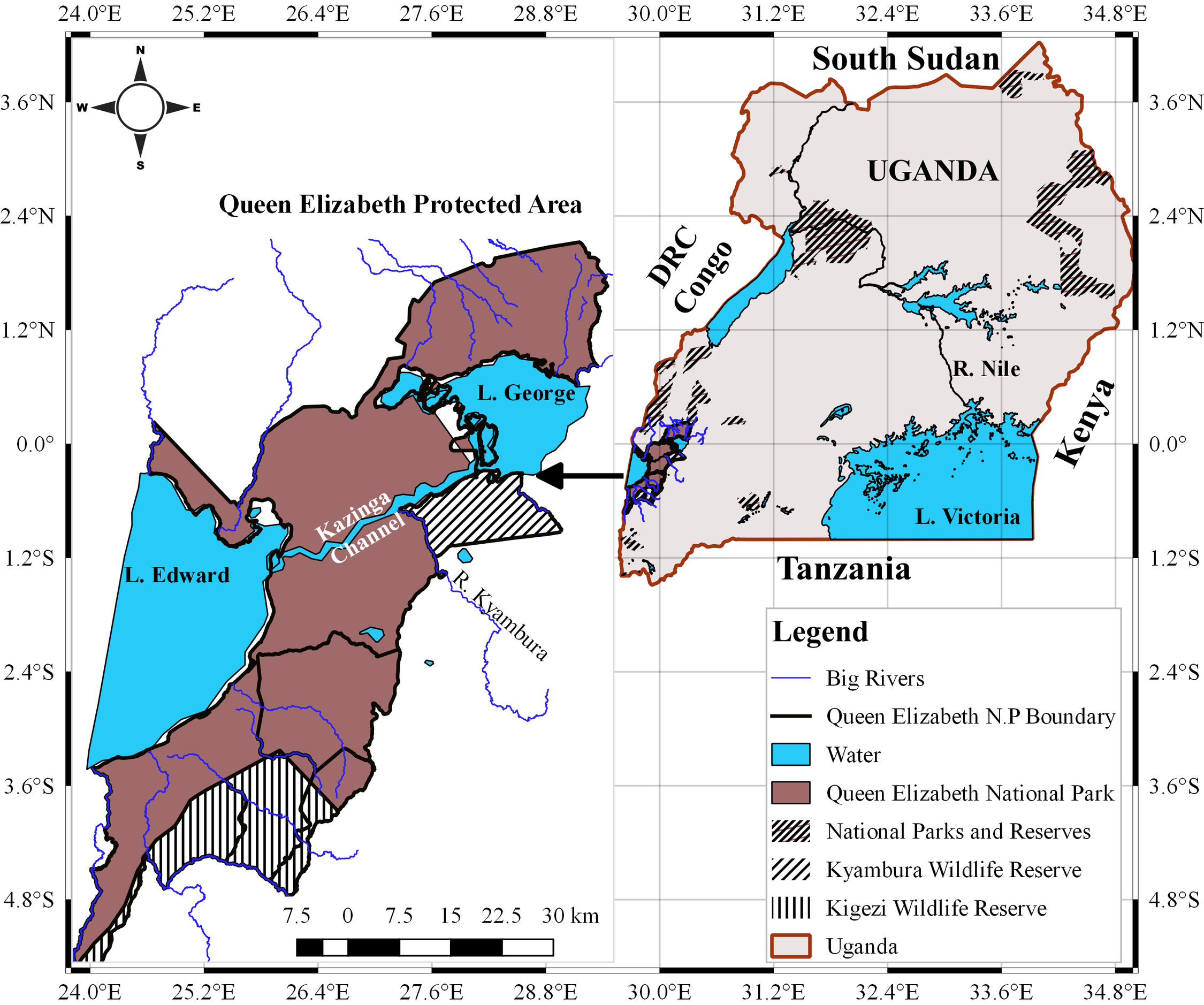

Supplement: S1 Fig — comprises of: 1) Queen Elizabeth National Park (1978 km2): brown coloured polygon; two adjacent wildlife reserves: 2) Kyambura (157 km2): diagonal hatched lines; and 3) Kigezi (330 km2): vertical hatched lines. There are two major fresh water lakes: L. George (northward) and Edward (southward) connected by 40km long natural Kazinga channel; the area is drained by a network of rivers. QEPA is a Man and Biosphere Reserve (MAB): there are 11 Fishing enclaves, with an estimated population of ≥300,000 people and approximately 30,000 livestock. (TIF) [file pone.0206922.s001.tif]

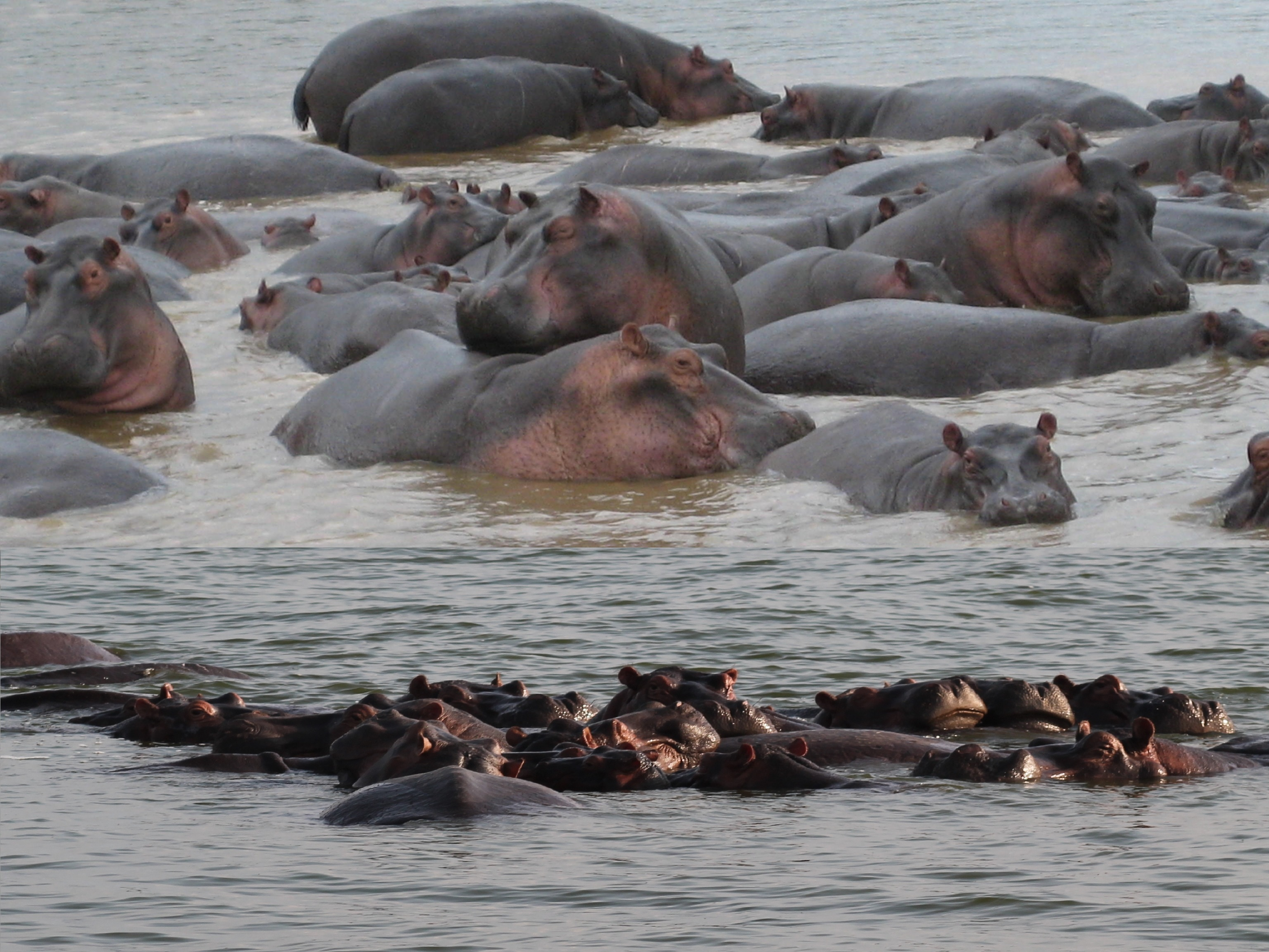

Supplement: S2 Fig — picture shows tight cohesiveness and congregation of hippos in enormous clumps with each individual resting and supporting its head and body on the other in shallow, calm, and low current waters (TIF) [file pone.0206922.s002.tif]

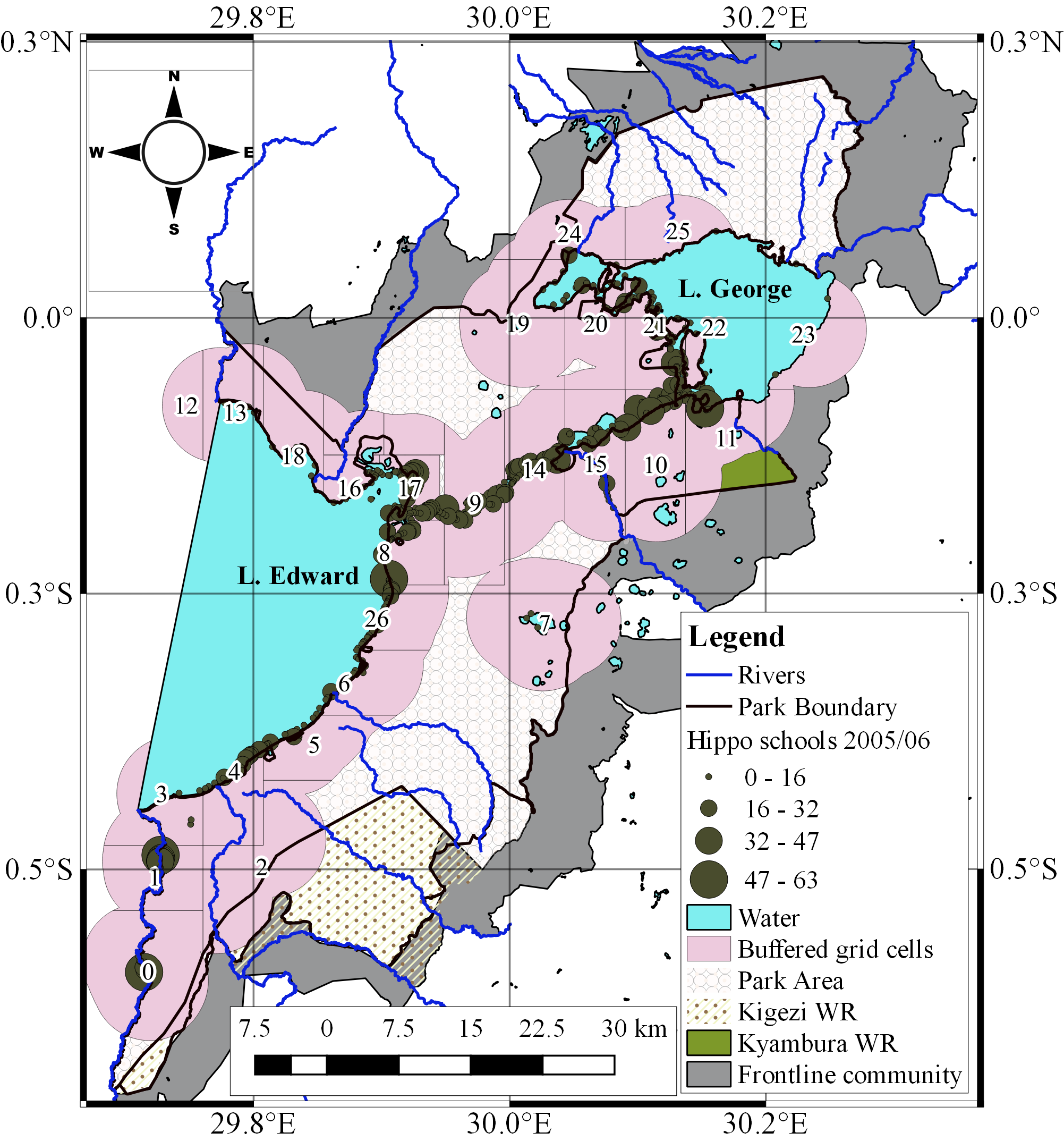

Supplement: S3 Fig — population census conducted from November 2005 to January 2006 in all water bodies within study area. Numbers against hippo schools (filled circles) indicate size of school at each location. School sizes varied from 1–63. Buffers represent average grazing distance for hippos of 6km from their respective schools. (PNG) [file pone.0206922.s003.png]

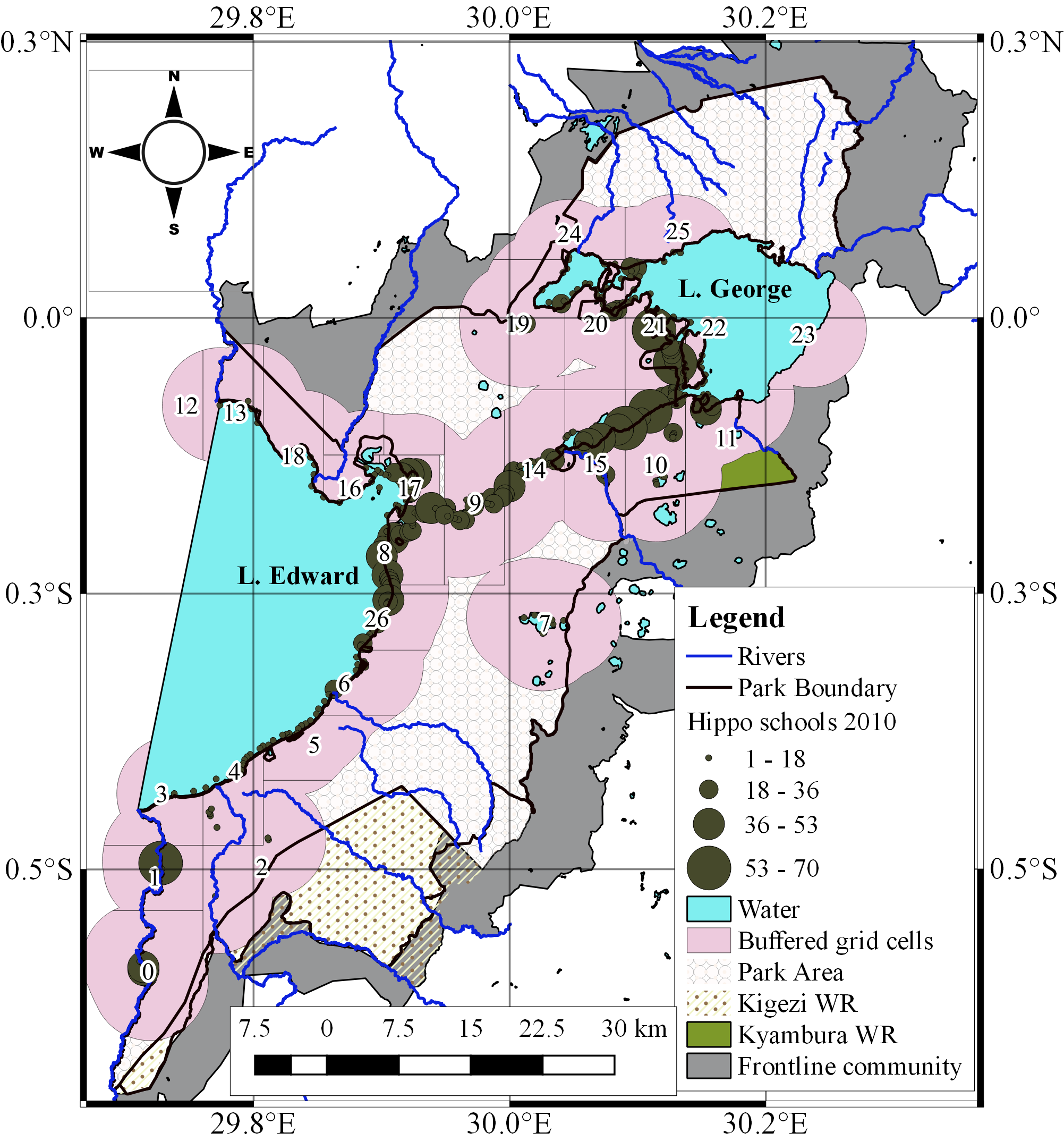

Supplement: S4 Fig — population census conducted from January to February 2010 in all water bodies within the study area. Numbers against hippo schools (filled circles) indicate size of school at each location. School sizes varied from 1–70. Buffers represent the average grazing distance for hippos of 6km from their respective schools. (PNG) [file pone.0206922.s004.png]
